# Evaluation of heavy metal contamination in copper mine tailing soils of Kitwe and Mufulira, Zambia, for reclamation prospects

**DOI:** 10.1038/s41598-022-15458-2

**Published:** 2022-07-04

**Authors:** Leonce Dusengemungu, Benjamin Mubemba, Cousins Gwanama

**Affiliations:** 1 School of Mathematics and Natural Sciences, The Copperbelt University, Kitwe, Zambia; 2 Africa Centre of Excellence for Sustainable Mining, The Copperbelt University, Kitwe, Zambia; 3 School of Natural Resources, The Copperbelt University, Kitwe, Zambia

**Keywords:** Ecology, Environmental sciences, Natural hazards, Solid Earth sciences, Mineralogy

## Abstract

Understanding the level of heavy metal contamination coupled with the assessment of environmental and human risks associated with mine waste dumpsites is an important step to initiating efficient measures for mine wasteland restoration, stabilization, and bioremediation. In the present study, concentration of the heavy metals; Copper (Cu), Cobalt (Co), Iron (Fe), Lead (Pb), Manganese (Mn), and Zinc (Zn) in soil from mine waste dumpsites around Kitwe (Sites: BM and TD26) and Mufulira (Site: TD10), Zambia, was assessed to determine the level of contamination, ecological risks, and progress made in reclamation. The mine waste dumpsites in the two towns are located in the vicinity of residential areas. Therefore, there is need to provide information for optimization of protocols for post-mining landscape in Zambia and elsewhere to limit soil, river, and groundwater contamination and to accelerate the restoration process . Mean values for soil pH, electrical conductivity, and organic matter varied between 5.9–8.4, 2534.8–538.6 μS/cm, and 0.90–2.75%, respectively. The mean concentrations of heavy metals of BM, TD26, and TD10 decreased in order of Fe > Cu > Co > Mn > Pb > Zn across all sites. However, the order of overall degree of heavy metal contamination computed using control soil as a baseline in BM, TD26, and TD10 was Cu > Co > Fe > Pb > Mn > Zn. The pollution load index was 0.355 at BM, 0.329 at TD26, and 0.189 at TD10, indicating high soil pollution at BM and TD26. The Potential Ecological Risk Index for all heavy metals tested at BM, TD26, and TD10 showed low ecological risk in the vicinity of the studied dumpsites. Furthermore, the present study also showed that the polluted soils around smelter sites and mine waste dumpsites are susceptible to dispersion by wind and water. Additionally, results from TD10 revealed that the initiated remediation of the tailings dam was somewhat successful. Finally, this study provided an updated status regarding the accumulation of heavy metals in mine waste dumpsites of Kitwe and Mufulira, Zambia and baseline information necessary to enhance post-mining landscape reclamation.

## Introduction

In recent years, a number of studies have reported a widespread concern about high levels of heavy metals from mining operations around mines, particularly in mine waste disposal sites^1–3^. These concerns have triggered assessment efforts of heavy metals in soils in mine waste dumpsites and around the mining areas as a potential first step in mitigating hazards to surrounding ecosystems and human health^4–6^. In developing countries such as Zambia, large areas have been affected by past and ongoing mining and smelting of minerals, resulting in chronic exposure to heavy metals by soil, water, plants, animals and humans^7,8^. It has been reported that human exposure to excess heavy metals might result in several health disorders, including skin lesions, cardiovascular disease, cancer of different organs, and reproductive defects^9,10^. For instance, some of these adverse effects have been reported in humans in Kabwe, Zambia, due to exposure to high Pb and Zn concentrations^11,12^. Similar health related problems have also been reported in animals. For example, in the north-eastern Transvaal, South Africa, a study revealed that cattle were dying due to chronic Cu poisoning^13,14^. Some heavy elements such as Cu, Zn, Fe, Mn and Pb are essential micronutrients for plant and animal growth. However, excess concentrations are harmful and considered pollutants in non-resistant plants where they limit plant growth^15^.

In the Copperbelt Province of Zambia, decommissioned mine tailings have resulted in extensive mine waste dumpsites that are contaminating water and soils, especially in Kitwe and Mufulira districts. Therefore, there is serious concern over the adverse effect of mine waste on the ecosystem, biodiversity, and health of people residing in the vicinity of these mine waste dumpsites^1,16–18^. Previous studies investigated the level of heavy metal pollution in soil, plants, and water and their probable impact on the environment in the Copperbelt Province^17,19–22^. Nevertheless, there are still gaps in the analysis of physicochemical characteristics of heavy metal concentrations around mine tailings dams and the evaluation of potential risk to humans and the environment.

It is hypothesized that the physicochemical characteristics of soil mine waste are associated with soil pollution^23^. These physicochemical characteristics determine the impacts of heavy metal pollution on the environment, especially in mining and high-intensity anthropogenic areas^8^. High heavy metal concentrations affect the biodiversity balance and activity of soil organisms, restricting soil organic matter decomposition and nitrogen and phosphorous mineralization^24,25^. However, there is considerable evidence suggesting that diverse soil organisms develop the capacity to resist high contamination of heavy metals, and their ability can facilitate restoring wastelands with high-quality biodiversity and to aid revegetation^26–28^. Establishing effective, inexpensive mine wasteland restoration protocols in Zambia and elsewhere would require a concrete understanding of the extent of heavy metal contamination and associated ecological risks, identification of candidate plant species for phytoremediation, and investigating the effect of organic amendments and the importance of soil microorganisms^29,30^. Therefore, supportive studies are required to address these concerns^1^.

The current study was aimed at filling some of these gaps. In the first place, we aimed to assess the pollution level of heavy metals in soils at tailings dams’ in the mine waste dumpsites of Kitwe and Mufulira, Zambia, and further assess progress of restoration in the tailings dams at Mufulira TD10, where restoration had been reported^31^. Finally, we used the potential ecological risk index (PERI) to examine the harmful effects of heavy metal contamination on surrounding human population and ecosystems, as well as evaluate the toxicity and ecological vulnerability associated with heavy metal contamination^32,33^. Previously, PERI has been used to provide evidence on the negative impact of heavy metal contamination on the environment^25,34,35^. The findings of the present study provide baseline data needed to plan revegetation strategies for mine waste dumpsites and tailings dams in Kitwe and Mufulira (which were decommissioned in the early 1980s and 1950s, respectively) and enable future assessment of potential ecological risks to humans and animals. Data-based reclamation of these mine tailings, would in-turn add to global success of mine dump reclamations.

## Materials and methods

### Study area

**Figure 1:**
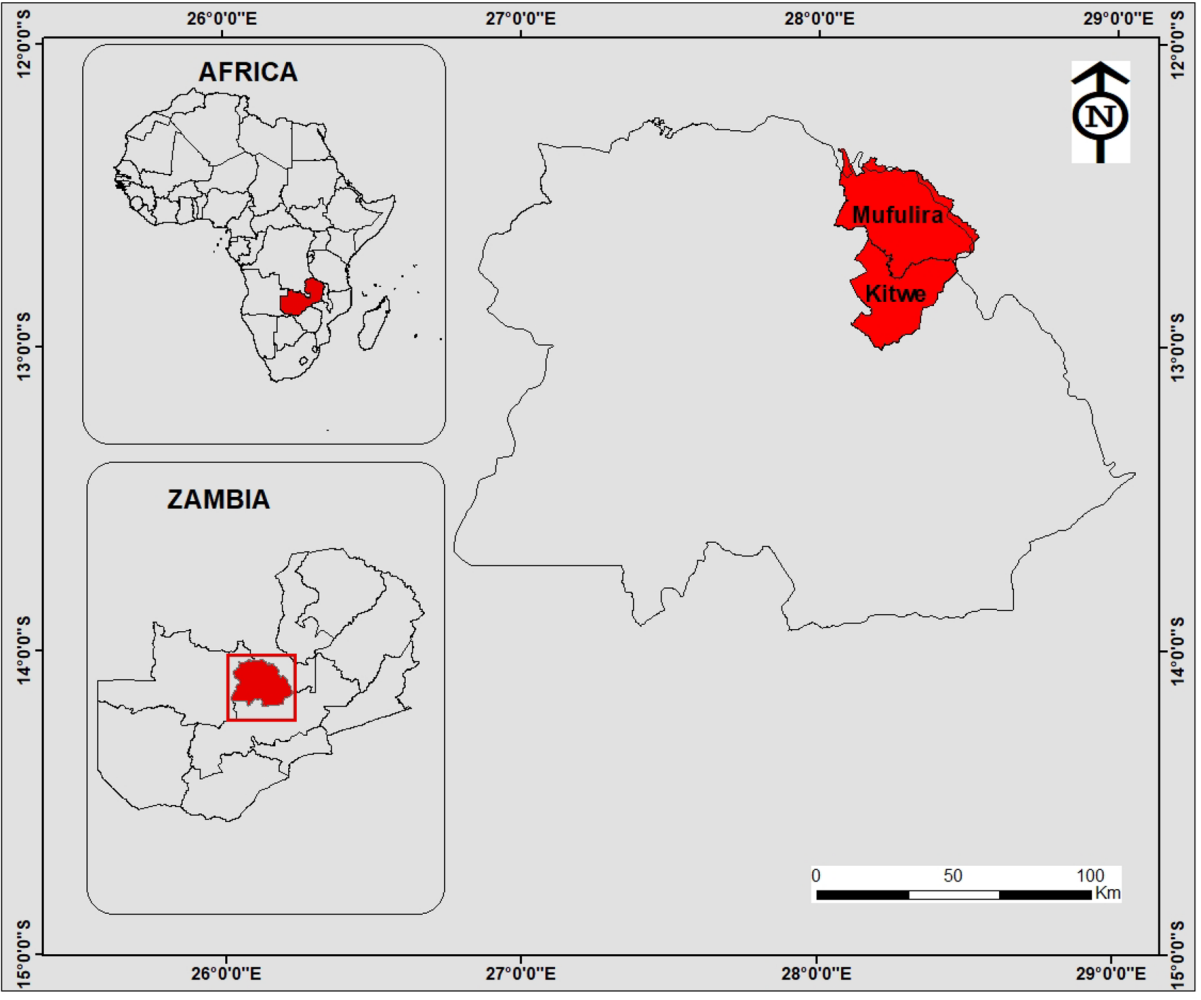
Geographical location of the study sites, Kitwe and Mufulira districts in red. The insert maps show the global position and the size of Zambia, highlighting the Copperbelt Province in red. Maps generated using ArcGIS 10.5 (ESRI, Redland California).

Two sites in Kitwe and one in Mufulira in the Copperbelt Province of Zambia were selected for this study (Fig. 1). Study sites were Cu and Co mining waste dumpsites and abandoned tailings dams. Nkana Slag Dumpsite (BM) is located at latitude 12°50′ S and longitude 28°12′ E, while Uchi Tailing Dam (TD26) is located at latitude 12°49′ S and longitude 28°13′ E. Mufulira Tailing Dam (TD10) is situated at 12°30′ S and 28°13′ E. According to the Environmental Council of Zambia^36^, the Copperbelt Province of Zambia has three seasons, namely; a rainy season (November to April), a cool- dry season (May–August), and a hot-dry season (August–November). Most of Zambia's terrain is a high plateau, and the country generally experiences a subtropical climate with average monthly temperatures ranging from 15.8 to 15.9 °C in June and July (the coldest months) to 26.3 °C in October (the hottest month)^36,37^.

### Sampling site description

#### The Black Mountain, Kitwe

**Figure 2:**
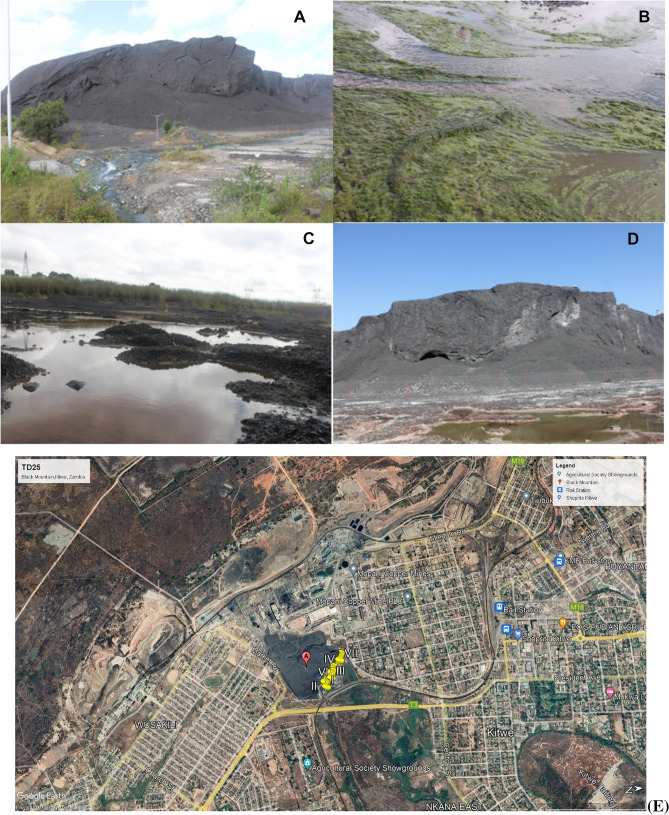
An overview of Nkana slag dumpsites in Kitwe. (**A**) River borders the Nkana Slag Dumpsite. Therefore, the erosion due to rain and wind might contaminate the water flowing from the river. (**B**) The algae colonization of the wastewater closer to the Black Mountain. (**C**, **D**) the wastewater, stagnant grasses, and trees surviving in the leftovers of the slag dump. Images: Leonce, June 2021. (**E**) An aerial photograph showing sampling points in yellow numbered I to VII at Nkana tailing dam location (BM) and surrounding area, Google Earth Satellite Image system, January 2022.

The Nkana Slag Dump in Kitwe (BM), also known as the Black Mountain “BM”, resulted from waste dumped from the copper smelter from 1931 until 2009. It is estimated to have 20 million tonnes of smelter slag with approximately 0.34 wt.% to 4.5 wt.% Co and an average of 1.2% Cu^38^. Seven mine dump locations were chosen for sampling to form a composite sample that was used to quantify the selected heavy metals. The selected sampling locations are shown in Fig. 2.

#### Uchi Tailing Dam, Kitwe

**Figure 3:**
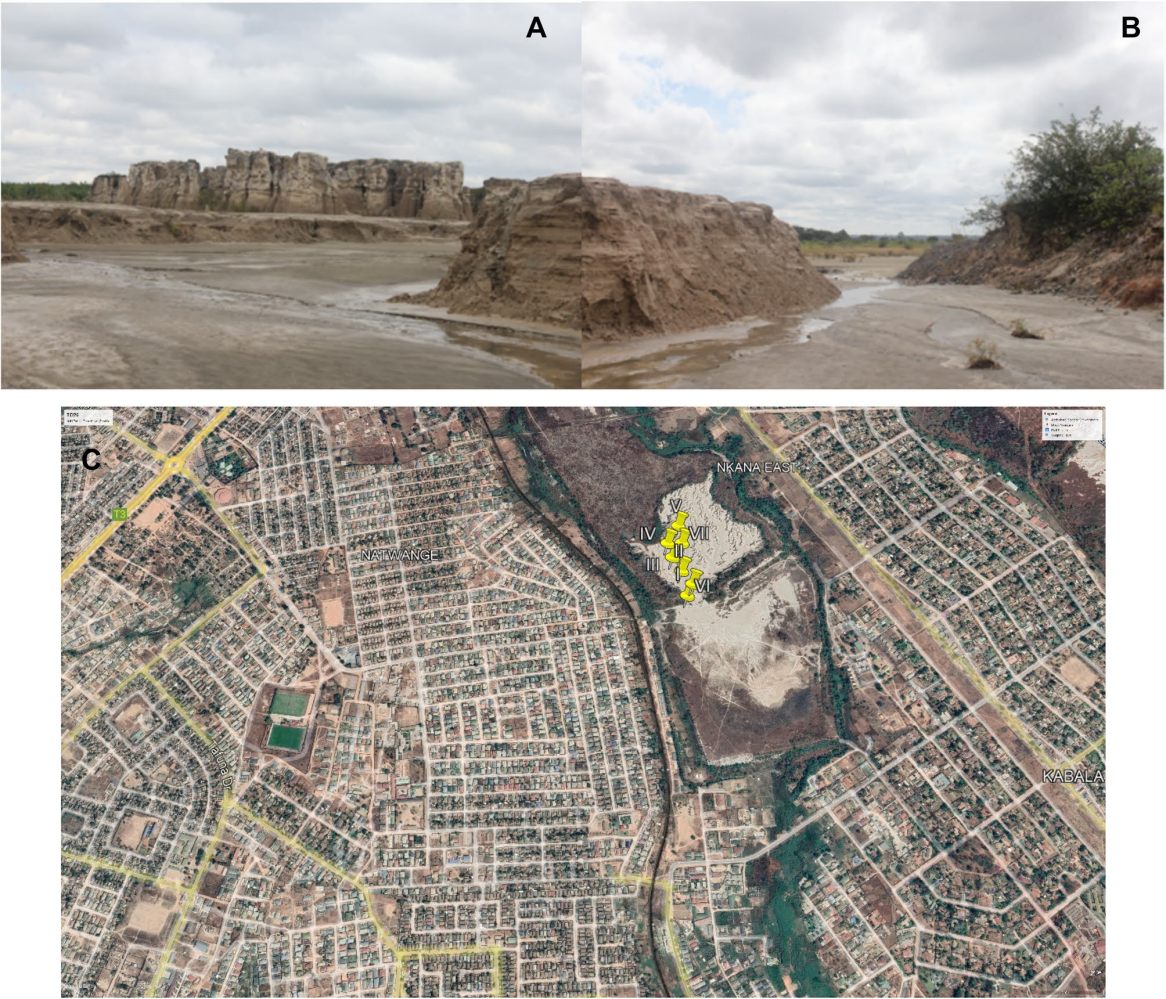
Uchi Tailing Dam (TD26). (**A**, **B**) Photographs showing compacted slurry on tailings, and gullies formed by water during the rainy seasons. The images: Leonce, June 2021. (**C**) An aerial photograph showing sampling points in yellow numbered I to VII at Uchi tailing dam location (TD26) and surrounding area, Google Earth Satellite Image system, January 2022.

Uchi Tailing Dam (TD26) is among the tailing dams that was used by Nkana Mine, resulting from the leftovers of Cobalt extraction from 1931 until 2009. Uchi Tailing Dam is located in the middle of residential areas and agricultural land (Fig. 3).

#### Mufulira

**Figure 4:**
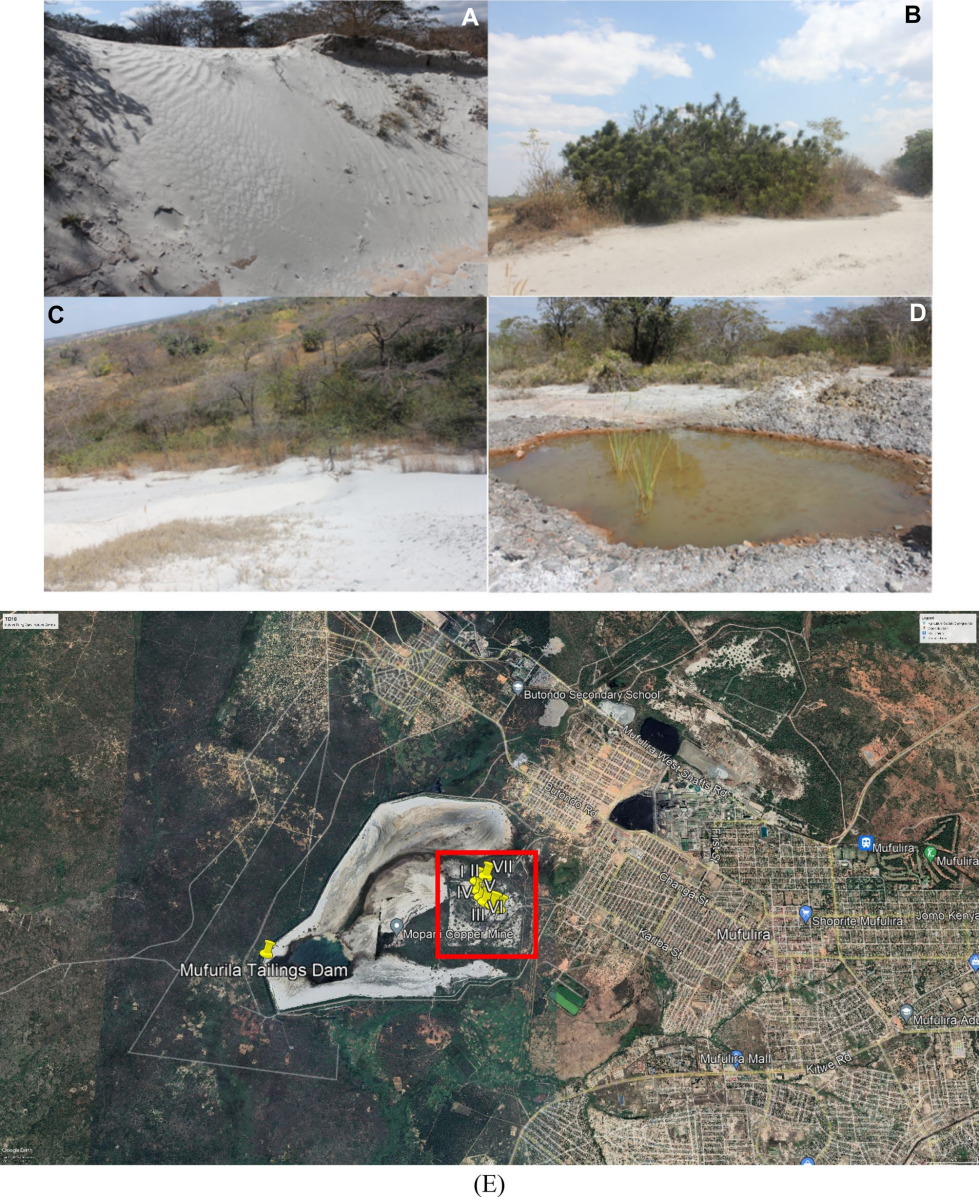
Mufulira TD10: (**A**) the copper tailings with some growing plants. (**B**, **C**) The ever-increasing plants in the tailing show the resistance capacity. (**D**) The wastewater dump formed near the tailings heaps at Mufulira TD10. Images were taken in June 2021. (**E**) An aerial photograph showing sampling points in yellow numbered I to VII at Mufulira tailing dam (TD10) location and the current area under reclamation and restoration in red square, Google Earth Satellite Image system, January 2022.

TD10 at Mufulira was established by Zambia Consolidated Copper Mines (ZCCM) as one of the tailing disposal facilities (TDFs) in an attempt to minimize the possible heavy metal contamination as a result of wind and water leaching/seepage into the surrounding surface and groundwater systems. The Mufulira tailing dam were decommissioned in 1988^31^. Seven locations at TD10 of Mufulira were chosen to quantify the selected heavy metal concentrations (Fig. 4).

### Sampling methodology and analysis

Soil samples were collected in June 2021 from a total of 26 randomly selected locations drawn from three sites; Nkana Slag Dump (BM), Uchi Tailing Dam (TD26), and Mufulira Tailing Dam (TD 10). The control soil samples were provided by Zambia Forestry and Forest Industries Corporation (ZAFFICO) in Kitwe. Soil samples were taken at every 100 m interval within a 50–100 m vicinity of the main area of the tailing dams. Approximately 500 g of a soil sample was collected from the top layer (0–25 cm) at each location using a trowel and placed in a polyethylene bag and was sealed. The collected samples were then placed in a cool box and were taken to the laboratory for analysis. After grinding the soils with a wooden mortar, samples were air-dried for 48 h on a laboratory bench and subsequently passed through a 2-mm sieve. The sediments samples were extracted using the conventional acid digestion method. Briefly, 1 g of each sample was placed in a 250 ml flask, followed by heating to 95 °C with 10 ml of 50% HNO_3_ without boiling. Following cooling, the sample was refluxed with repeated additions of 65% HNO_3_ until no brown odours were produced. The solution was then allowed to evaporate until it was reduced to 5 ml in volume. Following cooling, 10 ml of 30% H_2_O_2_ was progressively added without allowing any losses. The mixture was refluxed for 15 min at 95 °C with 10 ml of 37% HCl. The digestate was filtered through a 0.45 mm membrane paper, diluted to 100 ml with deionized water, and was kept at 4 °C until further analyses^39,40^.

### Physicochemical characteristics of mine waste soil

Physicochemical characterization was conducted on air-dried soil samples at the School of Natural Resources Soil Laboratory at Copperbelt University. Briefly, samples were suspended in CaCl_2_ (1:2.5 soil/CaCl_2_ (0.91 M) suspension) to measure pH and electrical conductivity (EC). The pH and EC of soil samples were measured using a combined electrodes laboratory pH/EC meter (Multi 3320_Xylem Analytics, Weilheim, Germany). The total organic carbon content of the soil samples was determined according to the Walkley and Black method^41,42^.

### Analysis of heavy metals in mine waste soil

The mine waste soil was analysed for Cu, Co, Fe, Mn, Pb, and Zn using a PinAAcle 900H flame/furnace Atomic Absorption Spectrophotometer (AAS: PerkinElmer Inc., Shelton, Connecticut, USA) equipped with Syngistix™ for AA software, version 4.0 at a commercial lab at Sable Zinc Kabwe Mines. The AAS PinAAcle 900H was first calibrated using standards for each metal. Every metal to be tested was read by employing specific lamps and parameters according to the metal (Table 1). Briefly, 1 g of soil samples was weighed and transferred to an Erlenmeyer flasks and 20 ml of Aqua Regia was added. The resulting suspension was boiled on the hot plate until appearance of nitrous fumes. Thereafter, the mixture was allowed to cool for five minutes and the Erlenmeyer flask was washed with 10 ml of distilled water, and 10 ml of HCl was also added. The mixture was boiled to dissolve the residue. Using a 100 ml volumetric flask, the sample was rinsed three times with distilled water. The washing was then transferred into another flask for further dilution with distilled water and homogenization by shaking. The total heavy metal content was calculated according to the method described in detail by^43^. The results were reported in ppm (parts per million). Each sample was digested and analysed three times for the purpose of observing the consistency in the results.

**Table 1:** Parameters set on the PinAAcle 900H flame/furnace Atomic Absorption Spectrophotometer (AAS).

Metal	Lamp specifications				Flame type
	Band width (nm)	Wavelength (nm)	Lamp current (mA)	Detection limits (ppm)	
Cu	0.7	324.75	15–25	0.001	Air
Co	0.2	240.73	15–25	0.006	Air
Fe	0.2	248.33	12–25	0.002	Air
Mn	0.7	279.48	12–25	0.002	Air
Pb	0.7	283.31	10–25	0.01	Air
Zn	0.7	213.86	8–25	0.002	Air

#### Quality assurance and quality control (QA/QC)

The quality assurance and quality control (QA/QC) procedures were examined by using standard reference materials, namely, the terrestrial global average (World recommended limit) for heavy metals in agricultural soil^44–47^ (Supplementary Table 4). To understand the level of contamination by Cu and Co, previous data obtained in Zambia and elsewhere were compared with the concentrations of heavy metals in this study. A total of 15 studies were selected and their findings were analyzed using Heatmap multivariate statistical techniques (Supplementary Table 4).Throughout all of the experiments, uncontaminated soil sample from ZAFFICO was used as the baseline.

### Data analysis

For data analysis, we used R Version 4.1.0 35 on R Studio version 1.4.1717 (Rstudio Inc., Boston, MA, USA) 36. The multiple comparison (pairwise comparison) test with R was applied to determine significant differences in mean heavy metal concentrations of different dumpsites. The pairwise comparison test was performed using the stats package^48^. The gglot2 package (Wickham, ^49^) was used to create a box plot of heavy metal data. The same software was used for Heatmap analysis to determine the sources of PERI due to different heavy metals in the sampled soil. The spatial sampling location was created using ArcMap 10 software (Environmental Systems Research Institute (ESRI), Redlands, CA, USA).

### Contamination factor (C.F.)

The contamination factor (C.F.), also known as the Geochemical Index (Igeo), is defined as the ratio calculated by dividing the concentration of each metal in the sampled soil by the baseline or background value (concentration in unpolluted soil) (Eq. 1). To determine the extent of contamination of the tailings dams, contaminant factor ( ${C}_{f}^{i}$ ), and the degree of contamination (${C}_{d}$) were calculated following Eqs. (1) and (2)^50^

1 $${C}_{f }^{i}=\frac{{C}_{0-1}^{i}}{{C}_{n}^{i}}$$

2 $${C}_{d}={\sum }_{i=1}^{n}{C}_{f}^{i}$$

where ${C}_{0-1}^{i}$ is described as the mean concentration of each metal in the substrate while ${C}_{n}^{i}$ is the concentration of the metal in unpolluted soil, which is the baseline value. In our case, it was the soil obtained from ZAFFICO plc. The contamination levels were presented according to scales described previously by Muller ^51^ and shown in Supplementary Table 1.

### Pollution load index (PLI)

The total PLI of all the sampling sites was calculated using the equation by Usero et al. ^52^(2000).

$${\text{PLI = }}\sqrt[n]{{CF_{1} \times CF_{2} \times CF_{3} \times \ldots CF_{n} }}$$

where *n* is the number of metals (*n* = 6 in the present study).

### Evaluation of potential ecological risk of heavy metals in mine waste soil

The following equation was used to compute PERI:

$$PERI=\sum_{i=1}^{n}Er =\sum_{i=1}^{n}Tr\times CF$$

where *Er* denotes the ecological risk factor of each heavy metal and *Tr* denotes the toxic response of each heavy metal. Supplementary Table 2 lists the grades used to measure ecological threats using PERI values, whereas Supplementary Table 3 lists the *Tr* and background values for heavy metal.

## Results

### Physicochemical characteristics of mine waste soil samples

The pH values of analysed soil samples a demonstrated a wide range of pH from alkaline to acidic soil. The mean pH of the mine waste soil at BM, TD26, and TD10 varied between 5.9 and 8.4, while the pH of soil samples from ZAFFICO plc (baseline control soil sample) varied between 5.5 and 6.7 (Table 2). The electrical conductivity of mine waste soil samples varied between 553 and 1765 μS/cm, whereas the mean electrical conductivity of baseline soil samples varied between 433 and 664 μS/cm (Table 2). The total organic carbon content of the mine waste soil from BM and TD26 varied between 0.9 and 1.6%, while the TOC content at TD10 varied between 0.4 and 1.9%, with the exception of one sample which had 2.2%. The baseline soil samples obtained from unpolluted soil ranged between 1.94 and 2.8% (Table 2).

**Table 2:** Mean ± S_e_ of pH, electrical conductivity and total carbon content of the mine soil samples collected from BM, TD 26 and TD 10.

Sites of sample collection	Variables tested (± S_e_)		
	pH ((CaCl_2_ (0.91 M))	Total organic carbon (TOC) (%)	Electrical conductivity (EC) (μS/cm)
Nkana Slag Dumpsite (BM)	7.4 ± 0.13	1.4 ± 0.18	25,348 ± 110.91
Uchi Tailing Dam (TD 26)	7.6 ± 0.36	0.9 ± 0.15	621.7 ± 31.79
Mufulira tailing dam (TD 10)	7.5 ± 0.16	1.6 ± 0.22	894.1 ± 108.72
ZAFFICO Plc (baseline)	6.0 ± 0.23	2.8 ± 0.34	538.6 ± 43.15

### Heavy metal concentrations in mine waste dumpsites

**Figure 5:**
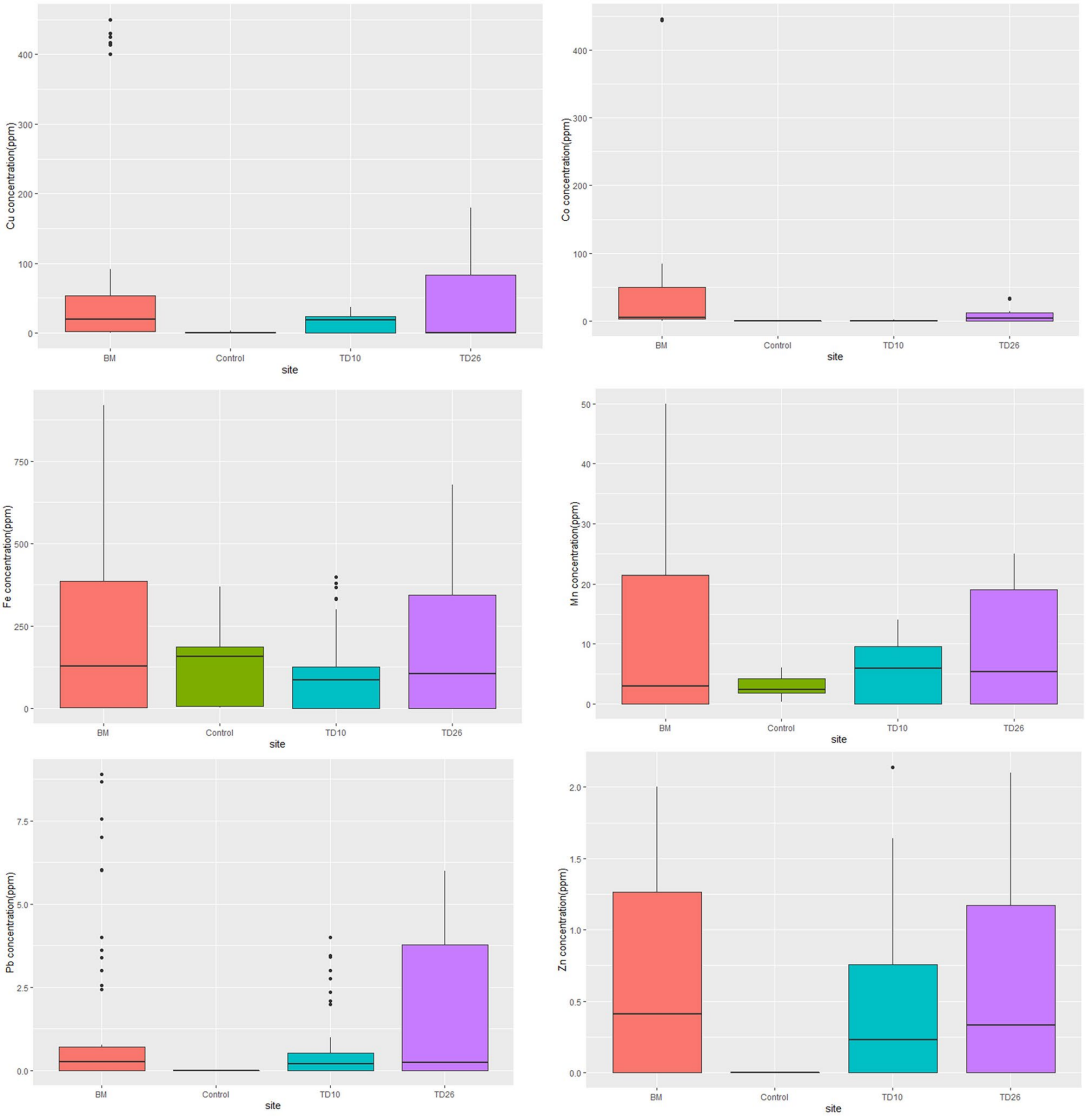
Boxplot of the heavy metal concentrations in soil samples of the three sampling sites, namely: BM, TD26, and TD10. The black solid line inside the box is the median value, and the black rhombus is the mean value; the black dots are individual samples.

The mean concentration of heavy metals between the dumpsites in Kitwe and Mufulira did not differ significantly (*p* value = 0.88 between BM and TD10 sites). Similarly, the p-value was 0.35 between BM and TD26 sites, while the p-value between TD26 and TD10 sites was 0.12. The mean concentration of heavy metals obtained from BM, TD26, and TD10 samples decreased in order of Fe > Cu > Co > Mn > Pb > Zn (Fig. 5). The dumpsites soil of Kitwe and Mufulira had a moderate quantity of potentially harmful heavy metals when considered in reference to control soil sample or when compared to the world recommended limits in agricultural soil^44,45^ (Supplementary Table 4),soil. Although the tested sites are relatively small and might not represent the entire mining area, they provide important insights into the extent of heavy metals contamination in the study sites (Fig. 5).

The mean concentration of Cu in soil samples was highest at BM site (138.9 ppm) while. Moderate levels of Cu were recorded for soil samples collected from TD26 (100.144 ppm) with the lowest level of Cu being recorded in soil samples collected at TD10 (22.46 ppm). For the baseline soil samples provided by ZAFFICO PLC, the concentration of Cu was 16 ppm (Fig. 5). Overall, the tailing dams in Kitwe (BM and TD26) had the highest concentration of Cu in comparison to the Cu concentration in Mufulira (TD10) (Fig. 5).

The mean concentration of Co was 19.5 ppm at BM, 13.7 ppm at TD26, and 1.2 ppm at TD10, and was present at a negligible concentration or below the detection limit of AAS instrument in control baseline soil (Fig. 5) (Supplementary Table 2). The Co concentration at BM was above 13 ppm and above the global terrestrial average in soil, while Co concentration at TD26 and TD10 was below the global terrestrial average^44^.

The soil obtained from BM had a higher Fe concentration compared with the soil from TD26 and TD10. The mean concentration of Fe in the soil from BM was 401.1 ppm (Supplementary Table 2). The mean concentration of Fe in soil from TD26 was 318.9 ppm (Supplementary Table 2) while the mean concentration of Fe in the soil from TD10 was lowest with 137.6 ± 102 ppm (Supplementary Table 2). However, the concentration of Fe was not significantly different among all tested tailing dams (Fig. 5).

The mean Mn, Pb, Zn concentration in the soil from BM was 14.8 ppm, 2.3 ppm, and 1.05 respectively, whereas the mean concentration of Mn, Pb, and Zn in the soil from TD26 was 16.1 ppm, 2.3 ppm, and 0.9 ppm, respectively. At TD10, the mean concentrations were Mn (7.8 ppm), Pb (0.9 ppm), and Zn (0.6 ppm). In the baseline soil, the Mn concentration was 3.8 ppm, while the Pb and Zn were not detected. When compared generally, the soil from Kitwe Tailing Dams (BM and TD26) had mean concentrations of each metal in a magnitude of 5–6 times more than to Mufulira Tailing Dam soil (TD10) (Fig. 5).

**Figure 6:**
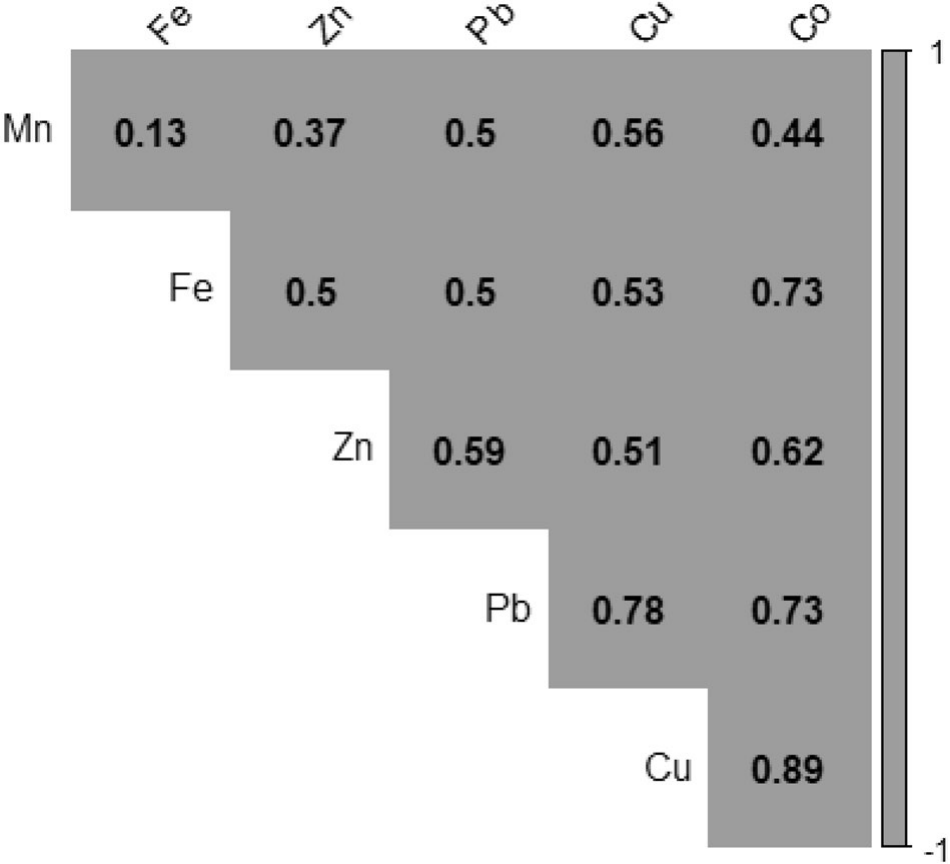
Pearson's correlation matrix for the heavy metals in mine soil based on sampling points across dumpsites, including the baseline.

To appreciate the correlation of heavy metal concentration between sites (BM, TD26, TD10), the Pearson's correlation coefficients were applied based on sample points across all sites (Fig. 6). Cu, Co, Fe, and Pb concentrations had a strong significant positive association with each other, suggesting a common origin. According to the scale of correlation coefficient (Supplementary Table 5), there were significant Mn-Cu, Fe-Cu, Fe-Co, Zn-Co, Zn-Cu, Pb-Cu, Pb-Co correlations indicating a common source and a close relation of the elements in the soil samples tested (Table 3).

**Table 3:** Contamination factor, overall degree of contamination, and pollution load index of different heavy metals at BM, TD26, and TD10.

Location	Contamination factor						The overall degree of contamination	PLI (Pollution Load Index)
	Cu	Co	Fe	Mn	Zn	Pb		
BM	6.04	1.51	2.16	0.03	0.01	0.09	9.84	0.31
TD26	4.35	1.06	1.71	0.03	0.01	0.09	7.26	0.25
TD 10	0.98	0.10	0.74	0.01	0.01	0.04	1.88	0.06
ZAFFICO plc	0.56	0.00	0.01	0.01	0.00	0.00	0.58	0.00

The heavy metal concentration in ZAFFICO soil was used as a baseline to calculate the contamination factor.

### The contamination factor and pollution load index of samples from BM, TD26, and TD10

The level of contamination by each tested heavy metal was determined using the baseline soil from ZAFFICO plc. The results showed that the highest contamination was by Cu and Co while lower levels of contamination were observed for Mn, Fe, Zn, and Pb at all the three dumpsites (BM, TD26, and TD10; Table 4). Specifically, the degree of contamination of Cu at BM was six times higher than the contamination found at TD10. The pollution load indices at BM, TD26, and TD10 were 0.31, 0.25 and 0.06, respectively (Table 3).

**Table 4:** Average contamination factor and contamination levels in soil samples.

Location	Heavy metal	Contamination Factor	Contamination level
BM	Cu	6.04	Extremely contaminated
BM	Co	1.51	Moderately contaminated
BM	Fe	0.01	Uncontaminated
BM	Mn	0.03	Uncontaminated
BM	Zn	0.01	Uncontaminated
BM	Pb	0.09	Uncontaminated
TD26	Cu	4.35	Strongly to extremely contaminated
TD26	Co	1.06	Moderately contaminated
TD26	Fe	0.01	Uncontaminated
TD26	Mn	0.03	Uncontaminated
TD26	Zn	0.01	Uncontaminated
TD26	Pb	0.09	Uncontaminated
TD10	Cu	0.97	Uncontaminated to moderately contaminated
TD10	Co	0.10	Uncontaminated to moderately contaminated
TD10	Fe	0.00	Uncontaminated
TD10	Mn	0.01	Uncontaminated
TD10	Zn	0.009	Uncontaminated
TD10	Pb	0.04	Uncontaminated
ZAFFICO plc	Cu	0.55	Uncontaminated to moderately contaminated
ZAFFICO plc	Co	0.00	Uncontaminated
ZAFFICO plc	Fe	0.01	Uncontaminated
ZAFFICO plc	Mn	0.01	Uncontaminated
ZAFFICO plc	Zn	0.00	Uncontaminated
ZAFFICO plc	Pb	0.00	Uncontaminated

### Analysis of PERI in mine waste dumpsite soil

**Figure 7:**
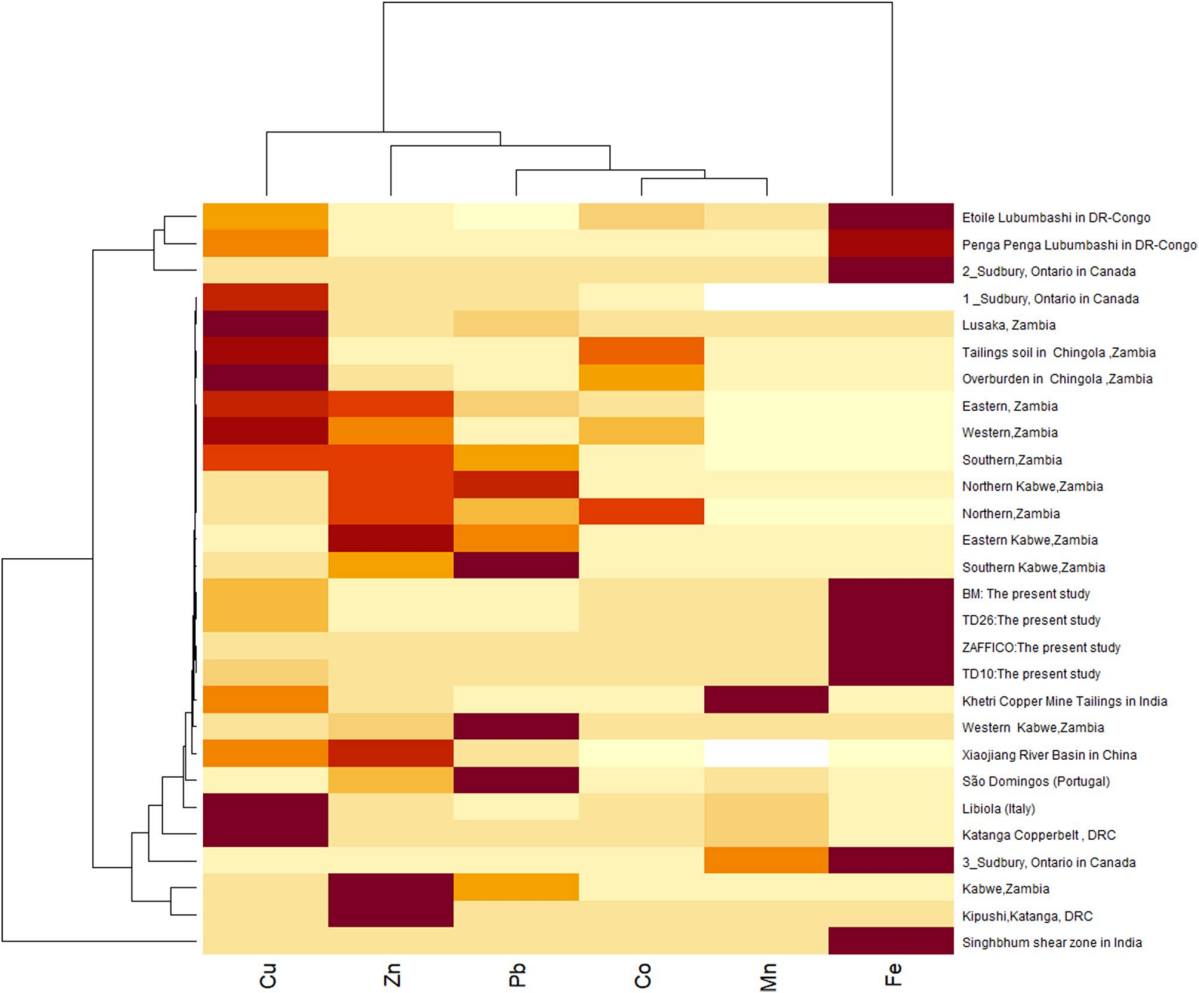
Heatmap analysis of heavy metals (Cu, Co, Fe, Mn, Pb, Zn) of mine soil in the present study with data from other studies in Zambia, DRC and elsewhere.

Descriptive statistics of ecological risk index data in soil are given in Table 5. The mean PERI values for heavy metals in soil were in the order of Fe (166.801) > Cu (38.38) > Co (18.46) > Mn (8.04) > Pb (0.96) > Zn (0.50). The PERI associated with each HM in mine waste soil analysed in the present study showed that all heavy metals had low ecological risk at the tailing dams of Kitwe and Mufulira (Table 6). On the other hand, the PERI associated with copper mine soil in other tailings dams of Zambia were at high ecological risk (Fig. 7). Other countries with various copper mine soil such as DRC, Italy, China, and Canada demonstrated very high ecological risk due to Cu and Co (Fig. 7).

**Table 5:** Descriptive statistics of potential ecological risk index data in mine waste soil of Zambia.

	Cu	Co	Fe	Mn	Pb	Zn
Min	0.00	0.00	0.00	0.00	0.00	0.00
Max	450.00	446	23,018.65	50	8.9	2.140
Mean	38.38	18.46	166.801	8.04	0.96	0.50
SE	0.069	7.50	5.67	0.85	0.15	0.27

**Table 6:** Calculated potential ecological risk index and toxic response coefficient level.

Metals	Ecological risk index (Er) of each HM	Toxic response coefficient	Grades of ecological perils
Cu	38.38	5	Low risk
Co	18.46	5	Low risk
Fe	166.801	1	Low risk
Mn	8.04	1	Low risk
Pb	0.96	5	Low risk
Zn	0.50	1	Low risk

The Heatmap of PERI of heavy metals in mine waste dumpsite soil (Fig. 7) indicated that Cu formed a distinct group with high variation in its concentration among the countries. Zn, Co, Pb and Fe also had separate group each with diverse variation in their concentrations among all analysed countries. The selected data from different studies were done on Cu and Co mine soil in other parts of world. The results show that the Copperbelt of Zambia and DRC formed a particular distinct Cu group.

## Discussion

The Copperbelt Province of Zambia extends from the border between Zambia and the south-central Democratic Republic of Congo (DRC) to nearly the centre of Zambia. Copper smelting in Zambia represents a significant source of soil pollution on the Copperbelt. Zambia's copper alone accounts for 6% of the global reserve and since 2009, Zambia mines between 600,000 and 800,000 tons of copper annually and 300,000 tons in the lean years^53^. On the other hand, the Democratic Republic of Congo mined 1.3 million metric tons of copper in 2020 alone^54^. The remaining copper deposits in Zambia and DRC are estimated at 6.52 and 30 million metric tonnes, respectively^55^. The 90 years-long history of mining in Zambia has resulted in approximately 791 million tons of tailings, 1,899 million tons of overburdened materials, 77 million tons waste rock, and 40 million tons of slags in Copperbelt Province alone^56^. Therefore, disposal sites for such enormous amount of mining/smelting and ongoing deposition of smelter stacks in operating mines pose a high ecological threat to surrounding areas^46,57^. Hence, detailed research was required to study the distribution of these particulates in the soil systems in the understudied Copperbelt Province of Zambia^38,58^.

### Mine waste soil physicochemical characteristics

The mean pH of the mine waste soil at BM, TD26, and TD10 was found to vary between 5.9 and 8.4, while the pH of baseline soil samples varied between 5.5 and 6.7. The reason the pH of some mine waste soil was acidic is due to the sulphidic ores from which copper is extracted using sulphuric acid. However, most soil samples tested from tailings dams had an alkaline pH which correlates with the previous findings at Mindolo tailing dams in Kitwe where the pH was 7.8. The alkaline pH in the mine waste soil can be attributed to the neutralization of acidic mine waste prior to effluent and tailings disposal^2^. The baseline soil from ZAFFICO plc were acidic and was in agreement with previous studies that established that most soil in the Copperbelt province including non-mining areas are generally acidic^59^. This is due to the advanced pedogenic process of weathering resulting from high rainfall and high temperatures in northern Zambia^59^. The observed high electrical conductivity in the mine waste soil is due to high salt content that characterises most dumpsites^60^. On the other hand, the observed low carbon content in the mine waste soil can be explained by the minimal plant growth on the study sites (and hence, lack of organic matter decomposition) due to high exposure to heavy metals in the topsoil of these areas^8,60^.This may make reclamation and restoration difficult because it is known that low organic matter in the soil does not encourage heavy metal absorption.

### The level of heavy metal accumulation, contamination factor, pollution load index and degree of contamination

Even though BM was decommissioned, the emerging new smelting technology has allowed the continuous smelting of Cu from the slags, leading to high concentration of Cu at BM^61^. This observation correlates with recent findings of an assessment of the topsoil in the vicinity of Nkana Smelter that highlighted the spatial distribution of metal/metalloid (As, Co, Cu, Pb, and Zn) in a 32 km^2^ zone around the smelter. It was established that Cu concentration was far above the global terrestrial average of 23 ppm^38^. This was attributed to the prevalent weathering and erosion effect in the area surrounding the Nkana Smelter and its surrounding tailing dam^38^. The weathering and erosion effects pose the risk of contaminating the soil and possibly water ecosystems of surrounding residential areas^18^. Given these observations, it is our considered view that the high amount of copper pollution necessitates an immediate response especially considering that dumpsites like TD26 are located in the middle of densely populated areas. Therefore, mitigating responses could include covering and sealing sulphidic mine waste, removing ore and waste dumps, designing and constructing a physical and chemical plant, constructing wetland environment, importing topsoil, and revegetation with local, metal-tolerant plant species. Additionally, efforts should be made to persuade local residents surrounding the tailing dams to produce crops elsewhere while restoration activities are taking place. Such approaches would help mitigate the health risks that the local community might be facing—such as respiratory complications and skin diseases arising from heavy metal poisoning—that might be less visible currently but may emerge over time. For example, a recent case study in the neighbouring city of Lubumbashi in the DRC revealed that chronic exposure to Cu and Co was a factor most significantly linked to birth abnonmalities^62^. This was particularly true for occupational mining exposure of paternal parents who constitute the bulk of the mining workforce. Equally, similar studies on human health should be conducted on the Copperbelt of Zambia, especially on the impact of copper mining waste disposed in tailing dams. Elsewhere, investigations revealed that Cu and Co were toxic to plants and animals and disrupted fishing, forestry, and agriculture activities hundreds of kilometers downstream of the mines and the tailing dams^63^.

The high Co levels observed in the present study can be explained by the fact that about 64% of the world's cobalt ore is located in Zambia's Copperbelt, together with the southern areas of the Democratic Republic of Congo (DRC). Cobalt is normally collected as a by-product of Cu processing and was thus increased in the ecosystem as observed in the present study (Supplementary Table 4).

The present study revealed that all three dumpsites tested high for Fe metal concentration. Fe was exceedingly high compared to other metals in soil obtained from slags of Kitwe dumpsites (BM), Uchi Tailing Dam (TD26), and Mufulira TD10 exceeding that of the baseline control soil. The iron was high due to the mining of Cu from a chalcopyrite (CuFeS_2_) deposit that is widely distributed is the mining regions. The present study results are in agreement with previous studies that investigate copper tailings as potential partial sand replacement in concrete making which found similarly high Cu, Fe, Mn, Ti, Mg, Al, Si, K, Ca, and S concentration at BM and TD26^64^. Results of those studies showed that Fe concentration was relatively higher than Cu concentration. However, iron pollution in the environment cannot be definitively connected to mining waste products alone as additional natural sources of iron must be considered^65^. In fact, soil iron toxicity is caused by the soil's naturally high accessible iron^66^. In comparison with international guidelines^8,67,68^, and ecological risk index findings, Fe concentrations determined in this study do not pose significant risks to humans or the environment.

The rainy season that commences from December until April resulting in vast volumes of water flowing throughout the study area also helps in the mobilization of heavy metals from mine waste dumpsites leading to contamination of local soil–water systems. Previous studies suggests that the presence of elevated concentrations of Cu and Co in the Kafue River network in the Copperbelt and the presence of slag glass and Cu–Fe–S intermediate solid solution in stream sediment was due to effects of Nkana Smelter and its surrounding tailing dams (BM and TD26)^21,69^. Other studies conducted on the Copperbelt made similar observations^1,30,70^. However, additional studies are still required to monitor and help mitigate the dispersal of heavy metal contaminants from mining and industrial activities to connected ecosystems such as the greater Kafue National Park ecosystem which is home to abundant biodiversity.

Description of the extent of metal pollution in the post-mining landscape in the Copperbelt Province is also vital to inform the contamination level, initiate restoration programs, study the progress in restoration, and determine the suitable plants for phytoremediation^71^. Indeed, given the level of contamination found in this study, reclamation activities are needed to correct or reduce the impact of the existing and potential hazards faced by areas bordering mining activities. We observed higher amounts of Cu and Co beyond the permissible global average. The maximum permissible concentration of Cu and Co in soil and sediments, is 20 ppm and 19 ppm, respectively^47,72^; while for Zn is 80–120 ppm, for Mn is 2000 ppm, 29,400 ppm for Fe and 50 ppm for Pb^8,67,68^. Compared to the global average metal concentration allowed in soil and sediment, BM had a slightly higher average concentration of Cu, Co, and minimal concentration of Fe, Mn, Pb, and Zn (Fig. 5) (Supplementary Table 4). The soil from TD26 showed a slightly higher average concentration of Cu and a lower concentration of Co, Mn, Fe, Pb, and Zn in comparison to the permissible global average (Fig. 5) (Supplementary Table 4). These findings show that the study areas (BM and TD26) were particularly contaminated with Cu and Co due to limited efforts in reclamation. The contamination factor at the BM tailing dam for Cu and Co were 6.04 and 1.51, respectively, indicating a high degree of contamination of Cu and minimal contamination of Co. Similar findings were observed at TD26, where the contamination factor of Cu and Co were 4.35 and 1.06, respectively. The other elements (Fe, Mn, Pb, and Zn) had a contamination degree of less than one at all sites, which denotes a low soil contamination level (Tables 3, 4).

Tailings dams are disposal facilities (TDF) that are often used to dispose of by-products from the mining process (water and other waste). They might cause a serious concern due to the possibility of metal mobilization on the TDFs through wind, leaching and leakage into the groundwater systems. Therefore, post-mining management of TDFs encourages the need to rehabilitate these areas to eliminate the environmental hazards concern, especially in areas where the dumpsites are closer to residential areas. The rehabilitation of tailing dams is necessary for soil restoration, biological processes, and vegetation establishment^73^. The observation at the BM shows high algae colonization (Fig. 2B) in the slag dump's surrounding wastewater. Therefore, it is expected that species of microorganisms have adapted to tolerate extreme metal concentration levels^27^. The low concentration of heavy metals at Mufulira TD10 can be attributed to the ongoing reclamation and restoration initiative using phytoremediation (Fig. 4)^31,74^. These positive results as indicated by low levels of metal contamination observed in this study are an indication of successful ongoing reclamation efforts that could be replicated at BM and TD26^31^. Remediation efforts using phytoremediation are recommended at all tested sites.

Excess concentrations of Cu and Co in plants cause symptoms such as chlorosis and necrosis, stunting, leaf discoloration, and root growth inhibition^75,76^. The slow growth of bush bean seedlings in response to adding 28 g/l of Fe to soil was previously reported^77^. Even though the Fe content at all mine wastes dumpsites studied was relatively higher than other metals, it was below the international standard Fe concentration allowed in agricultural soil. Therefore, we hypothesize that the stunted growth of plants and damping off of tree seedlings observed at different mining dumpsites in Kitwe and Mufulira as also reported by Festin et al.^74^, might be due to poor general soil condition, rather than Fe toxicity per se. In these environments, the toxic soil environment for plants includes extreme high osmotic potentials as reflected by high electrical conductivities revealed in the present study. Consequently, such high soil potentials do not only reflect mineral toxicities, but may also cause plant cell plasmolysis^68^. Additional studies are therefore required to determine effect of heavy metals on plant growth in the Copperbelt province, particularly in mine restoration sites. Such studies should be based on chemical form of the metals, because geochemical processes, valence changes, sorption/desorption, and bioprocesses play a significant role in the soil matrix environment. More research is also needed to understand the effect of heavy metals on biodiversity, particularly on microorganisms.

### Heatmap and principal component analysis of PERI of heavy metals mine waste dumpsites soils

Based on the PERI conducted for heavy metal contamination in mine waste soil, it can be concluded that heavy metal pollution is a potential risk in Zambia and the DRC (Fig. 6). The Heatmap assessment that included seven countries revealed that Zambia and the DRC constituted a unique group, which might be attributed to large variances in heavy metal PERI values. Heatmap assessment of copper mining activities in Zambia and the DRC showed pollution in topsoil. However, in the present study, the data revealed that the HM levels of Cu, Co, Fe, Mn, Pb and Zn in tailing soil were at considerably lower concentrations in comparison with other findings from tailings elsewhere in Zambia and other countries which explains the low ecological risk established in the present study. These findings imply that continued monitoring of mine wastes is required to restrict their potential release into the ecosystem.

The current study had some limitations. The fact that sampling procedures were done in duplicate in only a small area of the tailing dumpsites is the first constraint. As such, a more comprehensive assessment of the geographical distribution of soil pollution/contamination with heavy metals in the entire area of the tailing dams of Kitwe and Mufulira was not achievable. Therefore, additional studies are needed not only to assess the entire tailings dams’ area, but also to elaborate on the ecotoxicological and human health implications and to aid our understanding of metal transmission along terrestrial food chains on the Copperbelt province of Zambia.

## Conclusion and recommendations

The following conclusions were derived from this study:

1. Based on PERI assessment, it was observed that copper tailing dams remain one of the most serious environmental problems facing the Copperbelt of Zambia today.
2. High concentrations of Co, Cu, Fe, and medium concentrations of Mn, Pb, and Zn were observed in all the tailing dam sites, which is the main drawback for remediation. Minimal concentrations of all these elements were observed in the control sample in comparison with the tailing dams
3. Mine waste soil physicochemical characteristics (organic matter content, pH, and electrical conductivity) in the tested sites would not support promoting plant growth. Further research is required to assess more physicochemical properties such as total nitrogen, available phosphorus, potassium, magnesium, and cation exchange capacity (CEC).
4. The contamination factor and pollution load index led to the conclusion that the tailing dams have an excess of heavy metals. However, data from the TD10 locality showed that the initiated restoration for the tailings dam's remediation was somewhat succeeding.
5. According to PERI analysis, there is low ecological risk in the present study. However, the maximum PERI was observed in the data from elsewhere in Zambia and DRC which support that there is a high risk of contamination to humans and the environment.

Presently, however, there are few available data to assess the actual ecological hazard. Future research efforts should focus on improving our understanding of the impact of continuous exposure to Copper and Cobalt on humans and the environment. This will be critical when it comes to putting soil reclamation management plans into action.

In view of the key findings of the study, the following recommendations were made:

1. There is need for mine stakeholder institutions to develop and implement long-term policies, such as strict rules, that will result in excellent tailing dams restorations and recovery infrastructure, reducing waste and its health consequences,
2. Ensure that reclaimed soil and rivers are appropriately protected for agricultural operations.
3. To apply bioleaching technology to maximize the recovery of a significant amount of Cu, as well as other metals, can contribute to reduce the waste's environmental impact.

## Supplementary Information

Supplementary Information.

## Data Availability

The datasets generated and analysed during this investigation are not publicly available; however, they are available upon reasonable request from the corresponding author.
