# Author Correction: Evaluation of heavy metal contamination in copper mine tailing soils of Kitwe and Mufulira, Zambia, for reclamation prospects

**DOI:** 10.1038/s41598-022-26244-5

**Published:** 2022-12-22

**Authors:** Leonce Dusengemungu, Benjamin Mubemba, Cousins Gwanama

**Affiliations:** 1 School of Mathematics and Natural Sciences, The Copperbelt University, Kitwe, Zambia; 2 Africa Centre of Excellence for Sustainable Mining, The Copperbelt University, Kitwe, Zambia; 3 School of Natural Resources, The Copperbelt University, Kitwe, Zambia

Correction to: *Scientific Reports* 10.1038/s41598-022-15458-2, published online 04 July 2022

The original version of this Article contained a repeated error in the name of a sampling location, the Nkana Slag dumpsite, which was incorrectly given as “TD25” instead of “BM”. The error has been corrected in the sections Abstract, Materials and methods, Results, and Discussion. Additionally, Figures 2E, 5 and 7, Tables 2, 3 and 4, and the Supplementary Information 1 have been updated accordingly.

The original Article has been corrected.

