# Supplementary material for: Evaluation of heavy metal contamination in copper mine tailing soils of Kitwe and Mufulira, Zambia, for reclamation prospects
Source: Sci Rep. 2022 Jul 4;12:11283. doi: 10.1038/s41598-022-15458-2 (PMC9253116; doi:10.1038/s41598-022-15458-2)
Supplement: Supplementary file 1 — Supplementary Information. [file 41598_2022_15458_MOESM1_ESM.docx]

**Evaluation of heavy metal contamination in copper mine tailing soils of Kitwe and Mufulira, Zambia, for reclamation prospects**

Leonce Dusengemungu^1, 2*^, Benjamin Mubemba^3^, Cousins Gwanama^3^

^1^ School of Mathematics and Natural Sciences, The Copperbelt University, Kitwe, Zambia,

^2^. The Copperbelt University, Africa Centre of Excellence for Sustainable Mining, Kitwe, Zambia

^3^ School of Natural Resources, The Copperbelt University, Kitwe, Zambia

Corresponding author: dusengeleonce@yahoo.fr

**Supplementary material**

Table 1. Contamination factor (C.F.) for contamination levels in the soil

| Contamination Factor | Contamination Class | Contamination level |
| --- | --- | --- |
| >5 | 6 | Extremely contaminated |
| 4-5 | 5 | Strongly to extremely contaminated |
| 3-4 | 4 | Strongly contaminated |
| 2-3 | 3 | Moderately to strongly contaminated |
| 1-2 | 2 | Moderately contaminated |
| 0-1 | 1 | Uncontaminated to moderately contaminated |
| 0 | 0 | Uncontaminated |

Table 2.Grades of ecological risk indices used for assessment of ecological perils.

| Ecological risk index (Er) of each HM | Ecological risk indices (PERI) | Grades of ecological perils |
| --- | --- | --- |
| <40 | <150 | Low risk |
| 40-80 | 150-300 | Moderate risk |
| 80-160 | 300-600 | Considerable risk |
| 160-320 | >600 | High risk |
| >320 | - | Very high risk |

Table 3.Toxic response coefficient and background values of heavy metals.

| ppm | Cu | Co | Fe | Mn | Pb | Zn |
| --- | --- | --- | --- | --- | --- | --- |
| Toxic response coefficient^a^ | 5 | 5 | 1 | 1 | 5 | 1 |
| Background values^b^ | 23 | 13 | 29400 | 580 | 26 | 74 |

**a** ^1^**b**^2^

Table 4.The mean ±Se concentration (ppm) of heavy metals (Cu, Co, Fe, Mn, Pb, Zn) of mine waste soil collected from BM, TD26, TD10 and the baseline control soil samples obtained from ZAFFICO plc in the present study comparing with world recommend limit and various heavy metal concentration reported in literature.

| **Sites of Sample Collection** | **Heavy metal analyzed** | | | | | | **References** |
| --- | --- | --- | --- | --- | --- | --- | --- |
|  | **Cu** | **Co** | **Fe** | **Mn** | **Pb** | **Zn** |  |
| World recommended limits in agricultural soils | 23 | 13 | 29400 | 580 | 26 | 74 | ^2^ |
| Concentration range in surface horizons (worldwide) | 1/140 | - | 1000–100,000 | 7–9200 | 1.5−176 | 17–125 | ^3^ |
| Mean concentration in surface horizons (worldwide) | 14 |  | 45,000 | 437 | 25 | 64 | ^4^ |
| BM | 138.9±62.3 | 19.5±6.58 | 401.1±98.83 | 14.8±3.38 | 2.3±0.93 | \| 1.05±0.19 \| \| --- \| \|  \| \|  \| | Present study |
| TD26 | 100.1±38.5 | 13.7±6.76 | 318.9±131.56 | 16.1±3.97 | 2.31.17 | 0.9±0.29 | Present study |
| TD10 | 22.4±1.82 | 1.2±0.23 | 137.6±34.766 | 7.8±0.94 | 0.9±0.33 | 0.6±0.161 | Present study |
| Control (ZAFFICO plc) | 16±8.53 | 0 | 237.1±49.11 | 3.8±4.09 | 0 | 0 | Present study |
| Tailings soil in Chingola ,Zambia | 242.24 | 153.41 | - | - | 6.88 | 7.34 | ^5^ |
| Overburden  in Chingola ,Zambia | 146.76 | 54.2 | - | - | 2.07 | 7.93 | ^5^ |
| Eastern Kabwe | 8.5 | - | - | - | 130 | 231 | ^6^ |
| Northern Kabwe | 4.3 | - | - | - | 38 | 31.6 | ^6^ |
| Southern Kabwe | 7.9 | - | - | - | 160 | 72.2 | ^6^ |
| Western Kabwe | 58.2 | - | - | - | 759 | 106 | ^6^ |
| Lusaka, Zambia | 343 | 11 | - | - | 48 | 14 | ^7^ |
| Kabwe,Zambia | 572 | 46 | - | - | 7076 | 16991 | ^7^ |
| Eastern, Zambia | 37 | 11 | - | - | 13 | 32 | ^7^ |
| Western,Zambia | 36 | 13 | - | - | 5 | 22 | ^7^ |
| Southern, Zambia | 39 | 7 | - | - | 27 | 42 | ^7^ |
| Northern, Zambia | 29 | 94 | - | - | 52 | 99 | ^7^ |
| Katanga, DRC | 904 | 93 | - | - | 769 | 10182 | ^8^ |
| Katanga Copperbelt , DRC | 10320 | 990 | 4.5 | 1205 | 135 | 726 | ^9^ |
| Ajao, Nigeria | 2.09 | - | - | 0.012 | 0.004 | 0.01 | ^10^ |
| Zomba ,Malawi | 13.45 | - | - | - | 6.54 | 36.71 | ^11^ |
| Habo, Egypt | 26.19 | - | - | - | - | 71.39 | ^12^ |
| Kenya | 20.52 | - | - | - | 15.00 | 27.70 | ^13^ |
| Kunshan, China | 34.27 | - | - | - | 30.48 | 105.93 | ^14^ |
| Spain | 107.65 | - | - | - | 213.93 | 427.8 | ^15^ |
| America | 95.00 | - | - | - | 23.00 |  | ^16^ |
| Slovakia | 65.00 | - | - | - | 139.00 | 140.00 | ^17^ |
| USA | 48.00 | - | - | - | 55.00 | 88.5 | ^18^ |
| India | 1.2 | - | - | - | 0.95 | 28.24 | ^19^ |
| Iran | 9.62 | - | - | - | 5.17 | 11.5 | ^20^ |
| Singhbhum shear zone in India | 3314.4 | 63.8 | 64,695 | 1045 | 102.6 | 621.4 | ^21^ |
| Khetri Copper Mine Tailings in India | 230.6 | 13.5 |  | 469 | 21.6 | 87.6 | ^22^ |
| Xiaojiang River Basin in China | 696 | 41 |  |  | 236 | 1047 | ^23^ |
| Jiuhua copper mine in China | 3453 |  |  |  |  | 7244 | ^24^ |
| Penga Penga Lubumbashi in DR-Congo | 11,600 | 204 | 20,200 | 94.4 | 809 | 1,250 | ^25^ |
| Etoile Lubumbashi in DR-Congo | 14,200 | 6150 | 29,300 | 3,300 | 21 | 178 | ^25^ |
| Sudbury, Ontario in Canada | 300 | 34 |  |  | 46 | 59 | ^25^ |
| Sudbury, Ontario in Canada | 1,330 | 29 | 31,433 | 163 | 176 | 86 | ^25^ |
| Sudbury, Ontario in Canada | 373 | 37 | 12,933 | 6,610 | 46 | 52 | ^25^ |
| São Domingos (Portugal) | 21 | 21 | 10.06 | 393 | 3122 | 960 | ^26^ |
| Libiola (Italy) | 3543 | 91 | 16.54 | 639 | 20 | 364 | ^26^ |
| Ľubietová (Slovakia) | 3662 | 28 | 2.16 | 365 | 30 | 23 | ^26^ |
| Špania Dolina (Slovakia) | 1500 | 22 | 1.56 | 235 | 26 | 38 | ^26^ |
| Caporciano (Italy) | 7302 | 32 | 2.7 | 827 | 20 | 765 | ^26^ |
| Bor lies in Eastern Serbia | 25.97 |  | 112.24 | 41.01 | 7.88 | 10.51 | ^27^ |
| Pilar, Jaguar, Bahia Brazil | 433.4 |  | 434.4 | 53.4 |  | 5.7 | ^28^ |
| Pilar, Jaguar, Bahia Brazil | 606.7 |  | 168.1 | 44 |  | 5.2 | ^28^ |
| Chile | 418 |  |  |  | 46 | 160 | ^29^ |
| New South Wales, Australia | 3230 |  | 109100 | 1370 | 204 | 746 | ^30^ |

Table 5.The scale of Pearson´s Correlation Coefficient

| **Scale of Correlation coefficient** | **Value** |
| --- | --- |
| 0<r≤0.19 | Very Low correlation |
| 0.2≤r≤0.39 | Low correlation |
| 0.4≤r≤0.59 | Moderate correlation |
| 0.6≤r≤0.79 | High correlation |
| 0.8<r≤1.0 | Very high correlation |

**References**

1. Kumar, V. *et al.* A review of ecological risk assessment and associated health risks with heavy metals in sediment from India. *Int. J. Sediment Res.* **35**, 516–526 (2020).

2. Staniland, S. *et al.* Cobalt uptake and resistance to trace metals in comamonas testosteroni isolated from a heavy-metal contaminated site in the Zambian Copperbelt. *Geomicrobiol. J.* **27**, 656–668 (2010).

3. Kabata-Pendias, A. *Trace elements in soils and plants*. (CRC press, 2000).

4. Ajmone-Marsan, F. & Biasioli, M. Trace elements in soils of urban areas. *Water, Air, Soil Pollut.* **213**, 121–143 (2010).

5. Chileshe, M. N. *et al.* Physico-chemical characteristics and heavy metal concentrations of copper mine wastes in Zambia: implications for pollution risk and restoration. *J. For. Res.* (2019) doi:10.1007/s11676-019-00921-0.

6. Tembo, B. D., Sichilongo, K. & Cernak, J. Distribution of copper, lead, cadmium and zinc concentrations in soils around Kabwe town in Zambia. *Chemosphere* **63**, 497–501 (2006).

7. Ikenaka, Y., Nakayama, S. M. M., Muzandu, K. & Choongo, K. Heavy metal contamination of soil and sediment in Zambia. *African J. Environ. Sci. Technol. Vol.* (2010) doi:10.4314/ajest.v4i11.71339.

8. Mees, F. *et al.* Concentrations and forms of heavy metals around two ore processing sites in Katanga, Democratic Republic of Congo. *J. African Earth Sci.* **77**, 22–30 (2013).

9. Pourret, O. *et al.* Assessment of soil metal distribution and environmental impact of mining in Katanga (Democratic Republic of Congo). *Appl. Geochemistry* **64**, 43–55 (2015).

10. Emoyan, O. O., Peretiemo-Clarke, B. O., Tesi, G. O., Adjerese, W. & Ohwo, E. Occurrence, Origin and Risk Assessment of Trace Metals Measured in Petroleum Tank-farm Impacted Soils. *Soil Sediment Contam. An Int. J.* **30**, 384–408 (2021).

11. Mussa, C., Biswick, T., Changadeya, W., Mapoma, H. W. & Junginger, A. Occurrence and ecological risk assessment of heavy metals in agricultural soils of Lake Chilwa catchment in Malawi, Southern Africa. *SN Appl. Sci.* **2**, 1–8 (2020).

12. Abou El-Anwar, E. A. Assessment of heavy metal pollution in soil and bottom sediment of Upper Egypt: comparison study. *Bull. Natl. Res. Cent.* **43**, (2019).

13. Mungai, T. M. *et al.* Occurrences and toxicological risk assessment of eight heavy metals in agricultural soils from Kenya, Eastern Africa. *Environ. Sci. Pollut. Res.* **23**, 18533–18541 (2016).

14. Chen, F. & Pu, L. Relationship between heavy metals and basic properties of agricultural soils in Kunshan County. *Soils* **39**, 291–296 (2007).

15. Zimakowska-Gnoińska, D., Bech, J. & Tobias, F. J. Assessment of the heavy metal pollution effects on the soil respiration in the Baix Llobregat (Catalonia, NE Spain). *Environ. Monit. Assess.* **61**, 301–313 (2000).

16. Han, F. X. *et al.* Industrial age anthropogenic inputs of heavy metals into the pedosphere. *Naturwissenschaften* **89**, 497–504 (2002).

17. Wilcke, W., Krauss, M. & Kobza, J. Concentrations and forms of heavy metals in Slovak soils. *J. Plant Nutr. Soil Sci.* **168**, 676–686 (2005).

18. Jean-Philippe, S. R., Labbé, N., Franklin, J. A. & Johnson, A. Detection of mercury and other metals in mercury contaminated soils using mid-infrared spectroscopy. *Proc. Int. Acad. Ecol. Environ. Sci.* **2**, 139 (2012).

19. Prajapati, S. K. Heavy metal speciation of soil and Calotropis procera from thermal power plant area. *Proc. Int. Acad. Ecol. Environ. Sci.* **4**, 68 (2014).

20. Sayyed, G. & Sayadi, M. H. Variations in the heavy metal accumulations within the surface soils from the Chitgar industrial area of Tehran. *Proc. Int. Acad. Ecol. Environ. Sci.* **1**, 36–46 (2011).

21. Giri, S., Singh, A. K. & Mahato, M. K. Metal contamination of agricultural soils in the copper mining areas of Singhbhum shear zone in India. *J. Earth Syst. Sci.* **126**, 1–13 (2017).

22. Punia, A., Siddaiah, N. S. & Singh, S. K. *Source and Assessment of Metal Pollution at Khetri Copper Mine Tailings and Neighboring Soils, Rajasthan, India*. *Bulletin of Environmental Contamination and Toxicology* vol. 99 (Springer US, 2017).

23. Pu, W. *et al.* Effects of copper mining on heavy metal contamination in a rice agrosystem in the Xiaojiang River Basin, southwest China. *Acta Geochim.* **38**, 753–773 (2019).

24. Luo, X. S., Zhou, D. M., Liu, X. H. & Wang, Y. J. Solid/solution partitioning and speciation of heavy metals in the contaminated agricultural soils around a copper mine in eastern Nanjing city, China. *J. Hazard. Mater.* **131**, 19–27 (2006).

25. Narendrula, R., Nkongolo, K. K. & Beckett, P. Comparative soil metal analyses in Sudbury (Ontario, Canada) and Lubumbashi (Katanga, DR-Congo). *Bull. Environ. Contam. Toxicol.* **88**, 187–192 (2012).

26. Andráš, P. *et al.* Comparison of Soil Contamination at the Selected European Copper Mines. *Carpathian J. Earth Environ. Sci.* **16**, 163–174 (2021).

27. Filimon, M. N. *et al.* Potential ecological and human health risks of heavy metals in soils in selected copper mining areas—a case study: The bor area. *Int. J. Environ. Res. Public Health* **18**, 1–18 (2021).

28. Silva, F. A. *et al.* Determination of Hg in water by CVAAS using 2-aminothiazole modified silica. *Eclet. Quim.* **30**, 47–55 (2005).

29. Verdejo, J., Ginocchio, R., Sauvé, S., Salgado, E. & Neaman, A. Thresholds of copper phytotoxicity in field-collected agricultural soils exposed to copper mining activities in Chile. *Ecotoxicol. Environ. Saf.* **122**, 171–177 (2015).

30. Lottermoser, B. G., Ashley, P. M. & Lawie, D. C. Environmental geochemistry of the Gulf Creek copper mine area, north-eastern New South Wales, Australia. *Environ. Geol.* **39**, 61–74 (1999).
